# Comparison of non-invasive biomarkers faecal BAFF, calprotectin and FOBT in discriminating IBS from IBD and evaluation of intestinal inflammation

**DOI:** 10.1038/s41598-017-02835-5

**Published:** 2017-06-01

**Authors:** Yu Fu, Lingli Wang, Cheng Xie, Kaifang Zou, Lei Tu, Wei Yan, Xiaohua Hou

**Affiliations:** 1 Division of Gastroenterology, Union Hospital, Tongji Medical College, Huazhong University of Science and Technology, Wuhan, 430022 People’s Republic of China; 2 Department of Gastroenterology, Tongji Hospital, Tongji Medical College, Huazhong University of Science and Technology, Wuhan, 430030 People’s Republic of China

## Abstract

Faecal calprotectin and faecal occult blood test (FOBT) were widely used in the diagnosis and assessment of intestinal inflammation in inflammatory bowel disease (IBD). Recently we identified an excellent new biomarker B cell-activating factor (BAFF) for IBD. Here in this study we compared the efficacy of faecal BAFF, calprotectin and FOBT to find the “best non-invasive marker”. Results showed that for discriminating IBD from IBS, BAFF ≥227.3 pg/ml yield 84% sensitivity, 100% specificity, 100% positive predictive value (PPV) and 64% negative predictive value (NPV) while calprotectin ≥50 µg/g yield 76% sensitivity, 93% specificity, 97% PPV and 53% NPV. FOBT yield 65% sensitivity, 93% specificity, 97% PPV and 43% NPV. Combining BAFF with calprotectin tests yield 94% sensitivity, 93% specificity, 98% PPV, 81% NPV. Faecal BAFF level showed the stronger correlation with endoscopic inflammatory score as compared to calprotectin not only in UC (correlation coefficient [r] = 0.69, p < 0.0001 vs. r = 0.58, p < 0.0001), but also in CD (r = 0.58, p < 0.0001 vs. r = 0.52, p = 0.0003). Our results indicating that faecal BAFF is a promising non-invasive biomarker in IBD differential diagnosis and monitoring of intestinal inflammation.

## Introduction

Inflammatory bowel disease (IBD) including Crohn’s disease (CD) and ulcerative colitis (UC) are chronic idiopathic disorders with recurrent episodes of gastrointestinal inflammation. It is a common clinical challenge to differentiate irritable bowel syndrome (IBS) from IBD since both conditions share symptoms, such as abdominal pain and altered bowel habits. It was found that the IBS-like symptoms, despite the long-standing remission, remained in 59.7% of patients with CD and 38.6% of patients with UC^1^. To distinguish if the etiology is organic or functional, endoscopic evaluation is recommended. On the other hand, the correlation between clinical symptoms and objective measures of disease activity has mainly been poor, and active enteric inflammation can be present in patients without any symptoms^2, 3^. It is important for physicians to accurately understand the state of disease activity in each patient in order to treat and manage IBD properly. Though the examination of endoscopy is the gold standard for evaluation of intestinal inflammation, undergoing endoscopy is invasive and burdensome to patients, and is associated with a risk of perforation. Furthermore, it is difficult to perform endoscopic evaluation of mucosal lesion in clinical practice frequently. So finding alternative noninvasive biomarker or a set of markers which can accurately detect inflammation and monitor disease activity is necessary.

Faecal calprotectin is a neutrophil-derived calcium and zinc-binding cytosolic protein which is stable for up to one week at room temperature^4^. As a pioneer biomarker, it has been widely used in distinguishing inflammatory from functional bowel disorders and assessment of mucosal activity in IBD patients^5^. Occult intestinal blood loss frequently occurred in patients with IBD, and faecal hemoglobin could be used as a marker for mucosal inflammation in those patients. FOBT is widely used in clinical diagnosis since it could detect occult intestinal blood loss in a fast and cheap way with relative accuracy. B cell-activating factor (BAFF, also known as BLyS, TALL1, THANK or TNFSF13B), a member of the tumor necrosis factor (TNF) superfamily predominantly produced by myeloid cells (monocytes, macrophages, dendritic cells) and neutrophils, is critical for the maintenance of normal B-cell development and homeostasis^6^. Dysregulated expression and/or function of BAFF has been demonstrated to be associated with several human diseases, such as rheumatoid arthritis (RA)^7^, systemic lupus erythematosus (SLE)^8^, primary Sjogren’s syndrome (SS)^9^ and B cell malignancies^10^. For the first time, we recently reported on the performance of BAFF as a new biomarker in IBD^11^. It was shown that both serum and faecal BAFF serve as sensitive and specific markers for detecting IBD from IBS. Furthermore, the sensitivity and specificity of faecal BAFF is better compared with those of serum BAFF. But how does faecal BAFF perform compared with calprotectin and FOBT has not been investigated in IBD diagnosis and monitoring of inflammation.

The primary aim of this study was to evaluate faecal calprotectin, BAFF and FOBT as non-invasive markers in the diagnosis of IBD compared with the non-inflammatory condition, IBS. The secondary aim of this study was to compare the correlation of faecal calprotectin and BAFF with intestinal inflammation activity in patients with IBD.

## Material and Methods

### Patients

Consecutive patients with previously diagnosed IBD or with gastrointestinal symptoms suggesting IBD or IBS were recruited prospectively from two hospitals, the Union Hospital and Tongji Hospital of Tongji Medical College of Huazhong University of Science and Technology between May 2015 and Feb 2016. Healthy control (HC) (N = 26) were recruited from the Health Examination Center of Union Hospital.

Inclusion criteria were symptoms lasting for at least 3 months, complete colonoscopy with intubation of the terminal ileum including biopsies, age 18–70 years, informed consent, faecal samples collect within 2 days before colonoscopy (before the bowel preparation).

Exclusion criteria included incomplete colonoscopy, history of HIV infection, having infectious colitis within 6 month or microscopic colitis, history of colorectal surgery, colorectal cancer and regularly taking nonsteroidal anti-inflammatory drugs before their enrolment. Patients who were unable to provide a faecal sample were also excluded.

The diagnosis of newly onset IBD was prospectively established based on symptoms and preliminary examinations during outpatient visit and then verified by standard clinical, radiological, histological, and endoscopic criteria after admission. The diagnosis of IBS patients was based on the Rome-III criteria, no alarm symptoms, normal colonoscopy and histology manifestation. As for healthy control, all participants should be free of symptoms and having a normal clinical examination and abdominal ultrasonography. For CD patients with small intestinal lesions detected by radiological examination, balloon-assisted enteroscopy (BAE) was performed to get a clear observation of affected intestine. This study was approved by the Ethics Committee of Tongji Medical College, Huazhong University of Science and Technology. Informed consent was obtained from all participants and all methods were performed in accordance with relevant guidelines and regulations.

### Classification of the Severity of Disease

Clinical disease activity and endoscopic inflammation activity were determined in IBD. For clinical activity, the Crohn’s Disease Activity Index (CDAI)^12^ was used for CD patients and the Mayo Score^13^ for UC. For endoscopic inflammation activity, the Simplified Endoscopic Activity Score for Crohn’s Disease (SES-CD)^14^ was used in CD. The mucosal status was assessed according to the Mayo endoscopic subscore^13^ at each segments of the colorectum (cecum and ascending colon combined, transverse colon, descending colon, sigmoid colon, and rectum) in UC patients,. Each segment was scored as 0, normal or inactive disease; 1, erythema, decreased vascular pattern and mild friability; 2, marked erythema, absent vascular pattern, friability and erosions; and 3, spontaneous bleeding and ulceration in accordance with the MES. The maximum Mayo endoscopic subscore (MES) in the colorectum and the sum of Mayo endoscopic subscore (S-MES) in the five colonic portions ranging from 0 (no inflammation) to 15 (severe and extensive inflammation) was evaluated for analysis.

### Faecal extraction

The stool samples were collected freshly and stored at −80 °C until analysis. Faecal samples were weighted and reconstituted in extraction buffer (Calprotectin ELISA kit provided) to obtain a final concentration of 500 mg/ml and homogenized with an electric homogenizer (Tissue Lyser-24, Shanghai jingxin Industrial Development company, China) for 5 min twice to get a homogenous faecal suspension. After centrifuge at 12,000 rpm for 15 minutes at 4 °C, the supernatants (faecal extracts) were collected and stored at −80 °C until the measurement of calprotectin and BAFF. There is no overlap with the samples we previously published.

### Faecal Assays

The following commercial ELISA kits were used: BÜHLMANN faecal calprotectin^TM^ ELISA kit, Quantikine Human BAFF/BLyS/TNFSF13B Immunoassay and Faecal Occult Blood Test kit. All laboratory tests of faecal markers were performed blindly. The ELISA procedures were completed according to the manufacturer’s instructions and results were read on a microtiter plate reader (Tecan Infinite F50) at the absorbance of 450 nm. If the levels of BAFF or calprotectin reached the upper limit, samples were diluted further and measured again to obtain a quantitative value.

The BÜHLMANN faecal calprotectin^TM^ ELISA kit was purchased from BÜHLMANN LABORATORIES AG, Switzerland. According to the pre-experiment, lower range procedure with working range 10–600 µg/g was used after diluted the sample according to the manufacturer’s instructions. The intra-assay and inter-assay coefficient variability of this assay were 4.7% and <1.5%, respectively.

The Quantikine Human BAFF/BLyS/TNFSF13B Immunoassay was provided by R&D Systems, Inc, USA & Canada. BAFF was measured in the faecal extraction with the concentration of 500 mg/ml. The quantitative range was between 62.5 and 4,000 pg/ml. The intra-assay and inter-assay coefficient variability of this assay were ranging from 3.4% to 6.5% and from 10.0% to 11.6%, respectively.

Faecal Occult Blood Test kit was obtained from Baso Diagnostics, China, with the detection limitation of 50 µg(Hb)/g(stool).

### Endoscopic Assessment

Polyethylene glycol solutions were used for bowel preparation before endoscopic workup according to the standard protocol. All the patients received colonoscopy (CF-Q260, Olympus, Tokyo, Japan), while CD patients with small intestine lesions underwent BAE (SIF-Q260, Olympus, Tokyo, Japan) according to the results of computed tomography enterography (CTE). The endoscopic examinations were performed by experienced endoscopists who scored the intestinal inflammation activity blinded of the faecal marker results.

### Statistical Analysis

Statistical analysis was performed with SPSS 13.0. The results of numeric data were summarized by median (interquartile range, IQR). The levels of calprotectin and BAFF in different groups were analyzed by Mann-Whitney U-tests since the data were not normally distributed. The test characteristics are given as sensitivity, specificity, positive and negative predictive value (PPV, NPV), and overall accuracy. Receiver operating characteristic (ROC) curve was constructed to determine the best cut-off value and sensitivity and specificity of BAFF measurements as a diagnostic test. The cut-off of calprotectin chose 50 ug/g as the kit defined. The comparisons of AUC were analyzed by Z-analysis using software Medcalc 15.2.2. Associations between levels of faecal BAFF or calprotectin and disease severity or inflammation score were analyzed by Spearman’s rho correlation coefficient (r) for nonparametric correlations. A p value of <0.05 was used to define statistical significance.

## Results

### Patient Characteristics

Colonoscopies were performed and stool samples were collected from a total of 146 participants including 44 CD, 49 UC, 27 IBS and 26 healthy controls. Characteristics of enrolled patients are summarized in Table 1. In aspect of the disease location, 8 patients with CD had a history of ileum disease, 27 ileocolonic disease, and 9 colonic disease. In the group of UC patients, 14 of the patients had proctitis, 17 had left-sided colitis, and 18 had pancolitis. There were 17(38.6%) CD patients with a CDAI up to 150 (defined as clinical remission) and 27(61.4%) patients with a CDAI more than 150 (defined as clinical activity). Five patients (10.2%) with UC were in clinical remission (defined as ≤2 points). Five patients (10.2%) had mild disease (3–5 points), 27 patients (55.1%) moderate (6–10 points), and 12 patients (24.5%) severe disease (11–12 points) (Table 1).

**Table 1:** Baseline characteristics of study patients.

	CD	UC	IBS	HC
Patients				
Total	44	49	27	26
Age, Median (IQR)	28.5 (24.0–36.8)	39.0 (33.5–47.5)	34.0 (27.0–40.0)	32.0 (27.0–43.3)
Gender(F/M)				
Male	28 (63.6%)	31 (63.3%)	18 (66.7%)	15 (57.7%)
Female	16 (36.4%)	18 (36.7%)	9 (33.3%)	11 (42.3%)
Extent				
Ileocolitis	27 (61.4%)	—	—	—
Colitis	9 (20.5%)	—	—	—
Ileitis	8 (18.2%)	—	—	—
Proctitis	—	14 (28.6%)	—	—
Left-side colitis	—	17 (34.7%)	—	—
Pancolitis	—	18 (40.7%)	—	—
Disease behavior				
Stricturing	17 (38.6%)	—	—	—
Penetrating	7 (15.9%)	—	—	—
Inflammatory	20 (45.5%)	—	—	—
Clinical Disase activity				
CDAI*	182.0 (125.0–313.5)	—	—	—
Mayo Score*	—	8.0 (5.0–10.5)	—	—
Endoscopic activity				
SES-CD*	15.0 (9.3–20.8)	—	—	—
Mayo Endoscopic Score*	—	2.0 (2.0–3.0)	—	—
Therapy				
5-ASA	12 (27.3%)	26 (53.0%)	—	—
Glucocorticoids	12 (27.3%)	21 (42.9%)	—	—
Immunosuppressor	5 (11.4%)	2 (4.1%)	—	—
Anti-TNFa	15 (34.1%)	—	—	—

Median (IQR)for continuous variables; IQR, interquartile range; HC, healthy controls; IBS, irritable bowel syndrome; SES-CD, simplified endoscopic score for Crohn’s disease; CDAI, Crohn’s Disease Activity Index.

### Test characteristics of Faecal Markers

The test characteristics (median, IQR) of the quantitative assays for faecal markers are demonstrated in Table 2, making comparisons among healthy controls, IBS, and IBD patients. Faecal BAFF and calprotectin were significantly elevated in IBD patients compared with healthy controls (BAFF: P < 0.0001; calprotectin: P = 0.001). Statistically significant differences in faecal BAFF and calprotectin concentration were found between IBD and IBS (BAFF: P < 0.0001; calprotectin: P = 0.002), whereas no differences were observed in faecal BAFF and calprotectin content between IBS patients and healthy controls (BAFF: P = 0.976; calprotectin: P = 0.1004). These results indicated that BAFF and calprotectin were significantly elevated in IBD patients comparing with health control and IBS patients (Table 2).

**Table 2:** Test Characteristics of Faecal BAFF and Calprotectin.

		HC	IBS	IBD	HC vs. IBS HC vs. IBD IBS vs. IBD
BAFF (pg/ml)	Median	157.8	179.7	559.8	P = 0.976
					P < 0.0001
	IQR	85.1–189.9	97.9–190.8	226.6–1235.5	P < 0.0001
Calprotectin (µg/g)	Median	18.3	16.5	95.0	P = 0.1004
					P = 0.001
	IQR	15.5–24.6	14.5–19.7	52.6–467.6	P = 0.002

HC, healthy controls; IBS, irritable bowel syndrome; IBD, inflammatory bowel disease. IQR interquartile range.

### Performance of single faecal marker in discriminating IBS from IBD

**Figure 1:**
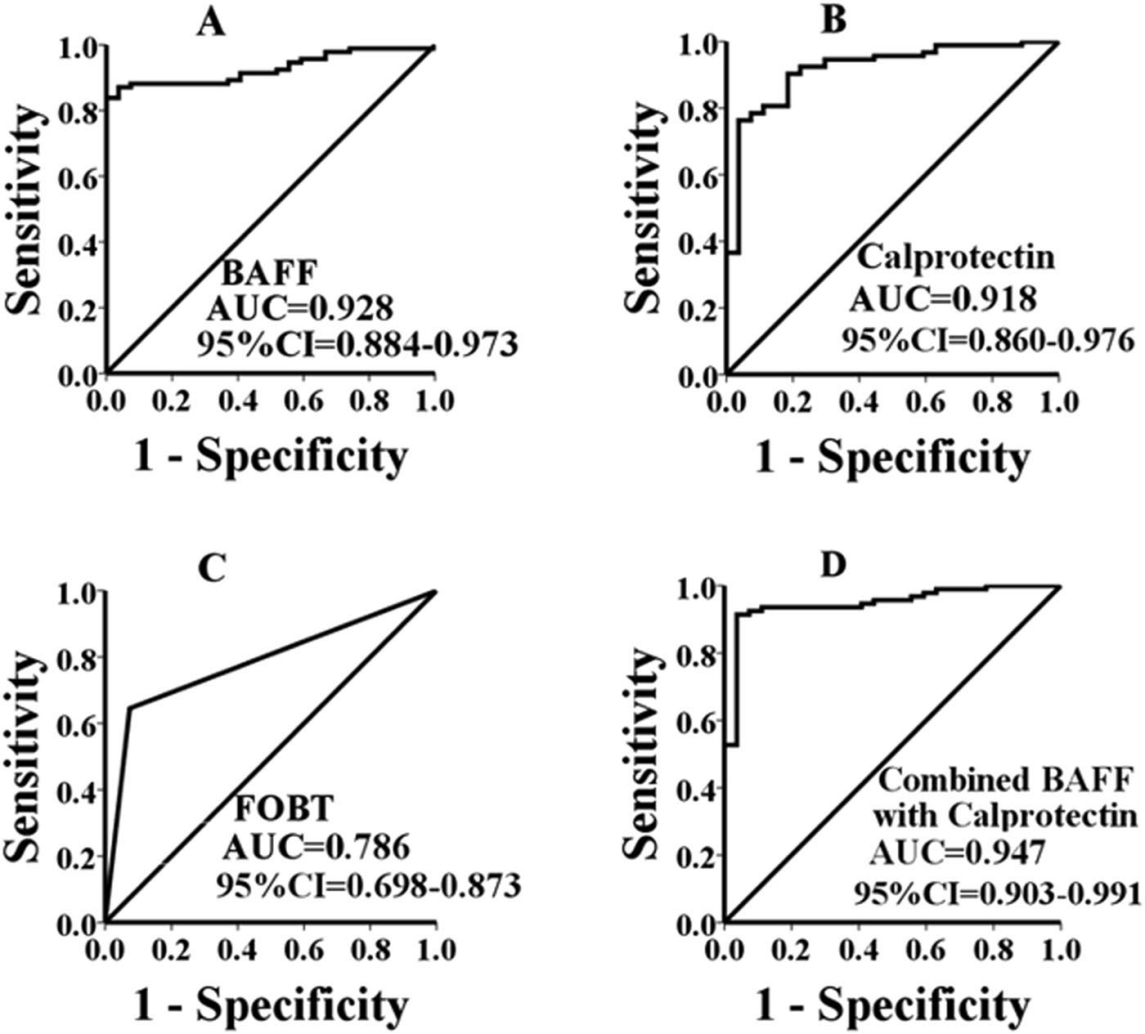
Receiver operating characteristic (ROC) curve of faecal BAFF, calprotectin, FOBT, and combined faecal calprotectin with BAFF in discriminating IBD from IBS with the area under the curve (AUC) of 0.928 (95% CI 0.884–0.973), 0.918 (95% CI 0.860–0.976), 0.786 (95% CI 0.698–0.873) and 0.947 (95% CI 0.903–0.991), respectively.

According to the ROC curve for faecal BAFF (Fig. 1A), the optimal cutoff value of 227.3 pg/ml was used to discriminate IBD form IBS, which showed a sensitivity of 84%, a specificity of 100%, a positive predictive value (PPV) of 100%, and a negative predictive value (NPV) of 64%. The cutoffs of calprotectin and FOBT were provided by the manufacturer. The global cutoff point of faecal calprotectin was 50 µg/g (sensitivity 75%, specificity 93%, PPV 97%, NPV 53%). FOBT (>50 µg/g) sensitivity and specificity to discriminate IBD from IBS was 66% and 93% (PPV and NPV of 97% and 45%, respectively) (Table 3). As showed in Fig. 1A and B, the AUC of faecal BAFF is numerically higher than that of faecal calprotectin, while no statistically difference was observed between them (P = 0.7677). The AUC of FOBT (Fig. 1C) were 0.786 which was much lower compared with faecal BAFF and calprotectin (p = 0.0003 and p = 0.0041). The results indicated that using the optimal cut off value test performance of faecal BAFF was superior to calprotectin and FOBT in differentiating IBS from IBD.

**Table 3:** Test Performance of faecal markers.

Test	Sensitivity	Specificity	PPV	NPV
BAFF				
IBD vs. IBS	84%	100%	100%	64%
CD vs. IBS	82%	100%	100%	77%
UC vs. IBS	86%	100%	100%	79%
Calprotectin				
IBD vs. IBS	76%	93%	97%	53%
CD vs. IBS	75%	93%	94%	69%
UC vs. IBS	78%	93%	95%	69%
FOBT				
IBD vs. IBS	65%	93%	97%	43%
CD vs. IBS	41%	93%	90%	49%
UC vs. IBS	86%	93%	95%	78%

PPV, positive predictive value; NPV,negative predictive value.

### Combined faecal markers performance in discriminating IBS from IBD

We were intrigued whether the combination of faecal BAFF and calprotectin would increase the test power of discriminating IBD from IBS. As is shown in Table 4, an improved sensitivity (94%) and NPV (81%) with slight impairment of specificity and PPV (100% to 93%, 100% to 98%, respectively) were observed when faecal BAFF and calprotectin were combined. The combined AUC increased slightly to 0.947 as compared with 0.928 for faecal BAFF and 0.918 for faecal calprotectin alone, but no significant difference was found between them (P = 0.4086 and P = 0.1801) (Fig. 1D). In addition, the combination of BAFF and FOBT or the combination of calprotectin and FOBT both led to higher sensitivity but lower specificity.

**Table 4:** Test Performance of Combined Faecal Markers.

Test	Sensitivity	Specificity	PPV	NPV
BAFF and Calprotectin				
IBD vs. IBS	94%	93%	98%	81%
CD vs. IBS	93%	93%	95%	89%
UC vs. IBS	94%	93%	96%	89%
BAFF and FOBT				
IBD vs. IBS	89%	93%	98%	71%
CD vs. IBS	86%	93%	95%	81%
UC vs. IBS	92%	93%	96%	86%
Calprotectin and FOBT				
IBD vs. IBS	85%	85%	95%	62%
CD vs. IBS	80%	85%	90%	72%
UC vs. IBS	90%	85%	92%	82%

PPV, positive predictive value; NPV, negative predictive value.

### Accuracy of Faecal Markers in discriminating IBS from IBD

The test accuracy of discrimination between IBS and IBD is shown in Table 5. In summary, faecal BAFF and calprotectin were highly accurate in distinguishing IBS from IBD (84% and 69%, respectively). These tests have advantages over FOBT (58%). When faecal markers were combined the accuracy was improved to discriminate IBS from IBD. The combination of BAFF and calprotectin resulted in an increased accuracy to 87% for discriminating IBD from IBS. The combination of BAFF and FOBT or the combination of calprotectin and FOBT improved the accuracy of discrimination IBS from IBD to a certain extent compared with using the faecal marker alone (82% and 70%). Therefore, the faecal BAFF have higher accuracy than calprotectin for the discrimination of IBD and IBS, meanwhile, the combination of faecal markers can improve the diagnosis accuracy.

**Table 5:** Accuracy of Faecal Markers and Combined Faecal Markers.

Test	IBD vs. IBS	CD vs. IBS	UC vs. IBS
BAFF	84%	82%	86%
Calprotectin	69%	68%	71%
FOBT	58%	34%	79%
BAFF and Calprotectin	87%	86%	87%
BAFF and FOBT	82%	79%	85%
Calprotectin and FOBT	70%	65%	75%

CD, Crohn’s disease; UC, ulcerative colitis; IBS, irritable bowel syndrome.

### Correlations Between faecal markers and Disease Activity Scores

**Figure 2:**
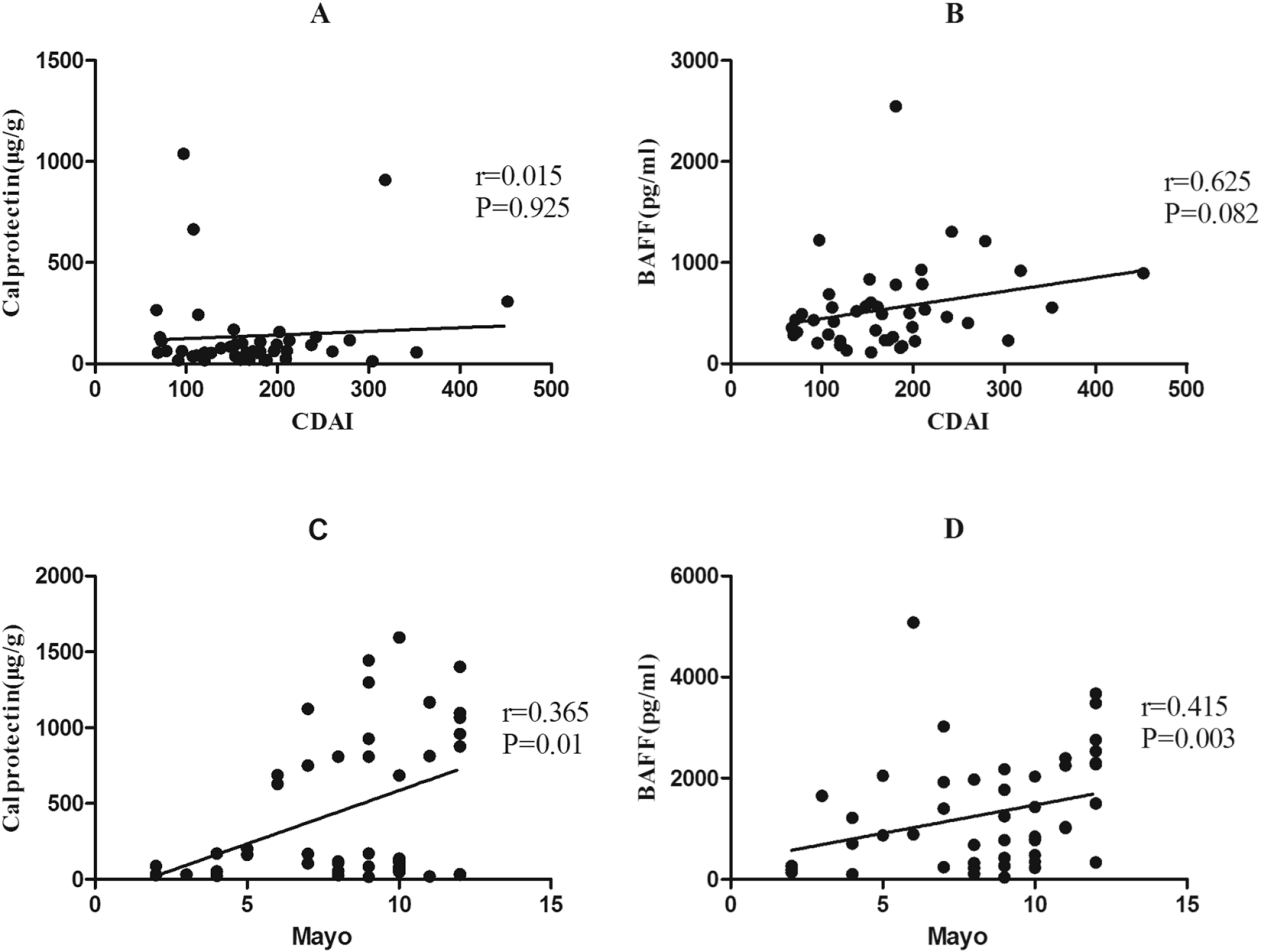
Analyses of correlations between faecal BAFF and calprotectin with disease activity scores for patients with CD (CDAI) and UC (Mayo score). (**A**,**B**) The faecal calprotectin and BAFF were uncorrelated with the CDAI (Spearman’s rank correlation coefficient [r] = 0.015, P = 0.925 and r = 0.625, P = 0.082, respectively). (**C**,**D**) The faecal calprotectin and BAFF were significantly correlated with Mayo Score (r = 0.365, P = 0.01 and r = 0.415, P = 0.003, respectively).

We were interested in whether there were significant correlations between faecal markers and disease activity indices. As shown in Fig. 2A and B, faecal BAFF and calprotectin were not found significant correlations with Crohn’s Disease Activity Index (r = 0.625, P = 0.082; r = 0.015, P = 0.925, respectively). There were significant but poor correlations both between faecal BAFF (r = 0.415, P = 0.003) and calprotectin concentrations (r = 0.365, P = 0.01) with clinical activity index in UC patients (Fig. 2C and D). According to the analysis, we revealed that faecal BAFF and calprotectin significantly correlated with disease activity scores in UC but not in CD.

### Correlations Between faecal markers and Endoscopic Findings

**Figure 3:**
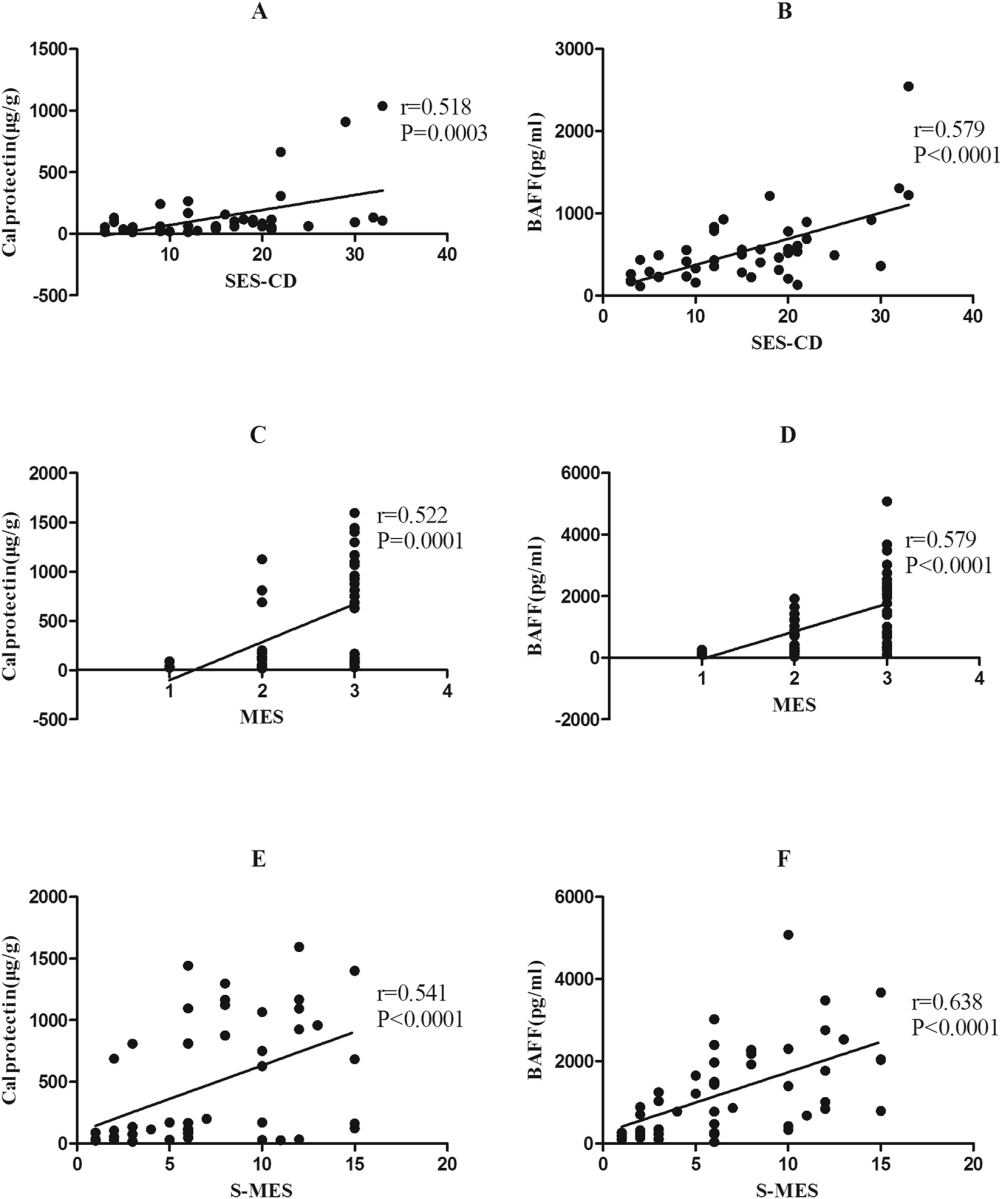
The correlations between faecal markers and the endoscopic inflammation score for patients with CD (SES-CD) and UC (MES and S-MES). (**A**,**B**) The faecal calprotectin and BAFF results were significantly correlated with SES-CD (Spearman’s rank correlation coefficient [r] = 0.518, P = 0.0003 and r = 0.579, P < 0.0001, respectively). (**C**,**D**) The faecal calprotectin and BAFF results were significantly correlated with MES (r = 0.522, P = 0.0001 and r = 0.579, P < 0.0001, respectively). (**E**,**F**) The faecal calprotectin and BAFF results were significantly correlated with S-MES (r = 0.541, P < 0.0001 and r = 0.638, P < 0.0001, respectively).

The data for endoscopic assessment of severity in CD and UC are presented in Table 1. The coefficient of correlation and the corresponding P-value among the BAFF, calprotectin, and endoscopic findings was illustrated. With regard to CD, Faecal BAFF and calprotectin demonstrated highly significant correlation with SES-CD (Spearman’s rank correlation coefficient r = 0.579, p < 0.0001; r = 0.518, p = 0.003, respectively), as shown in Fig. 3A and B. In UC patients, BAFF (r = 0.579, P = 0.0001) and calprotectin (r = 0.522, P = 0.0001) were significantly correlated with MES. Furthermore, BAFF (r = 0.638, P < 0.0001) and calprotectin (r = 0.541, P < 0.0001) demonstrated a strong correlation with S-MES, as shown in Fig. 3C–F. Correlations between faecal BAFF content and endoscopic inflammation indices were closer than that of faecal calprotectin both in CD and UC. Therefore, those results implied that BAFF concentration may be preferably in monitoring endoscopic inflammation.

## Discussion

Efforts have been made for decades to find a reliable non-invasive biomarker for the early detection and monitoring intestinal inflammation of IBD. Although a bunch of biomarkers such as pANCA, ASCA, lactoferrin, S100A12, granulocyte–macrophage colony stimulating factor, soluble CD14, and lipopolysaccharide-binding protein have contributed to our understanding of IBD pathogenesis, none is of sufficient effective to warrant clinical development as predictive biomarkers^15^. This is the first study to compare the efficacy of faecal BAFF with faecal calprotectin and FOBT for discriminating IBS from IBD and assessment of intestinal inflammation in IBD after we identified BAFF as a novel biomarker in IBD^16^. In this present study, our results demonstrated that faecal BAFF and calprotectin concentrations were significantly higher in IBD patients than that in IBS patients and healthy controls. Although no significant difference was found between the AUC of faecal BAFF as well as calprotectin for distinguishing IBD from IBS, the test performance of BAFF had an advantage over calprotectin and FOBT under the optimal cutoff value of 227.3 pg/ml, which yield a sensitivity of 84%, a specificity of 100%, a PPV of 100%, and a NPV of 64%. It means that faecal BAFF could be used as a high specific biomarker for differentiating IBD from IBS with a considerable sensitivity. Moreover, this study revealed that there was highly significant correlation of faecal BAFF with endoscopic inflammation severity, which were closer than the correlation between mucosal inflammation and widely approved faecal marker, calprotectin. Based on our study, faecal BAFF can be considered as a promising faecal marker used in discrimination IBD from IBS and in evaluation of mucosal inflammation.

Many patients with IBD present with abdominal pain and altered bowel habits in active stage or in clinical remission, which are also the common symptoms in patients with IBS. Therefore, it is a common clinical challenge for physician to differentiate IBS from IBD and endoscopic examination is recommended to correctly differentiate both. As is known to all, endoscopic examination is invasive, expensive and intolerable for some patients, potentially accompanied with a risk of perforation. In addition, clinical data demonstrated that the majority of patients with chronic abdominal discomfort did not find serious pathological changes through endoscopic examination^17^. Therefore, it is exceedingly necessary to find an alternative noninvasive biomarker which can accurately differentiate functional or organic disease and detect mucosal inflammation to avoid inappropriate examination.

As the best accepted surrogate marker, a cohort of studies previously have reported about faecal calprotectin testing, including separating IBD from IBS^18, 19^, differentiation between inactive and active forms of IBD^18–22^ prediction of clinical relapse in UC^23, 24^ and evaluation of mucosal inflammation^3, 25–28^. Several studies demonstrated that faecal calprotectin was capable of distinguishing IBD from IBS with the sensitivity ranging from 63% to 100%, the specificity from 79% to 93%^29–32^. These variations may result from the selection of different ELISA kit, cutoff values and different cohort of patients. Schoepfer *et al*.^18^ demonstrated that the faecal calprotectin and lactoferrin are highly more accurate for discriminating IBD from IBS than Hexagon-OBTI, blood leukocytes, CRP, and IBD Antibodies. In addition, the result of other articles showed the diagnostic overall accuracy of faecal calprotectin ranging from 0.56 to 0.96 for discriminating IBD from IBS^33^. In our study, the faecal calprotectin and BAFF were identified to succeed in discrimination IBD from IBS and the test performance of faecal calprotectin are in accordance with those studies. However, the calprotectin concentration was generally lower in the present study than in other studies. The reason may be that patients in clinical remission were included in our study. Moreover, some factors were reported to possibly influence the diagnostic capacity of biomarker tests, including patients with physical inactivity, obesity and ageing, high fiber intake and vegetable consumption and considerable day-today variability in some patients^34^. In present study, the AUC of faecal BAFF was 0.928(95% CI 0.884–0.973) for discriminating IBD from IBS, which is similar to our previous result of 0.933(95% CI 0.874–0.992)^11^. The sensitivity (84%) and specificity (100%) is also close to previous results (84% and 96%), although the optimal cutoff value was different between the present 227.3 pg/ml and previous 325 pg/ml. The variation may come from different batches of ELISA kits and cohort of patients.

It is interesting to know that combining faecal BAFF and calprotectin increased the sensitivity to 94% as compared to discriminate IBD from IBS with faecal BAFF (84%) or calprotectin (74%) alone. This may result from the different mechanisms of biomarkers involved in the intestinal inflammation in IBD. As a neutrophil-derived protein which constitutes 60% of neutrophil cytosolic protein^4^, faecal calprotectin reflected the level of acute inflammation cells infiltration in intestine, which was in the downstream of intestinal inflammation cascades in IBD. While BAFF is secreted mainly from myeloid cells including monocytes, macrophages and dendritic cells, acting as an important regulator of peripheral B-cell survival, maturation, immunoglobulin production and immunoglobulin class-switch recombination^35^, and reflecting the development of chronic inflammation more than acute. Moreover, our previous study showed that BAFF increased in serum in IBD patients compared with IBS and health controls and correlating well with diseases activity and the levels of IL-1β, TNF-α and C-reaction protein^11^, implying that BAFF takes an active role in the development of intestinal inflammation in IBD. So these two biomarkers reflect different aspects of intestinal inflammation in IBD.

Several studies have explored the association between faecal markers and clinical indices of diseases activity. In a large cohort of 164 CD patients undergoing colonoscopy, no significant association was found between the faecal levels of calprotectin and the CDAI scores^36^. Similar results were observed in our study for patients with CD. Meanwhile, faecal BAFF concentrations in CD patients also had no significant correlations with CDAI scores. These may result from the bias of subjective symptoms, since patients with IBS may have higher CDAI than patients with CD^37^. While in UC, significant correlations were manifested not only between faecal BAFF and Mayo scores (r = 0.415) but also between faecal calprotectin and Mayo scores (r = 0.365). Similar results can be seen in several previous studies, showing the significant correlations between faecal calprotectin and Mayo scores in UC^26, 38^. In terms of correlations between faecal markers and clinical indices of diseases activity, faecal BAFF is similar to faecal calprotectin, which is correlated with Mayo scores in UC but not with CDAI in CD.

Since we detected faecal BAFF and calprotectin levels in the same samples, we were able to compare the efficacy of faecal BAFF and calprotectin for accessing the severity of endoscopic inflammation in IBD. In CD, several studies have analyzed the significant correlation between faecal calprotectin levels and mucosal inflammation, with the correlation coefficients from 0.48 to 0.75 in CD^3, 26, 27, 39^. In our study, the correlation coefficient in CD(r = 0.518) was equivalent to previously published data. Besides the most used endoscopic indice of MES reflecting the severity of inflammation, our study also introduced the sum of MES(S-MES) in 5 colonic segments to evaluate the combination of severity and extent of intestinal inflammation in UC. Our correlation coefficient of 0.522 between faecal calprotectin and MES and 0.541 between faecal calprotectin and S-MES were in line with previous study ranging from 0.49 to 0.81 in UC^25–27, 40, 41^. Correlations of faecal BAFF to MES(r = 0.579) and S-MES(r = 0.638) were both higher than that of faecal calprotectin (r = 0.522 and r = 0.541, respectively). Furthermore, correlation of S-MES to faecal BAFF was stronger than that of MES implying that faecal BAFF could be a good indicator for overall evaluation of mucosal inflammation combining severity and extent. So, faecal BAFF have a better performance as compared with faecal calprotectin in evaluation of intestinal inflammation both in UC and CD.

Studies showed that the distribution of calprotectin in feces was even. RØseth AG *et al*. reported that the values of faecal calprotectin in randomly collected samples were similar to that in blended feces. The correlations between spot samples and the corresponding blended sample were rather strong [Pearson’s correlation coefficient(r) varied between 0.90 and 0.95]^4^. Hege TØn *et al*. found similar results that there was no significant difference in the mean faecal calprotectin levels measured in unblended and blended stools. The linear relationship between the mean measurements in blended and unblended feces was strong (r = 0.98)^42^. To determine whether BAFF is evenly distributed in feces, we tested contents of BAFF in random and the corresponding blended samples and found that there was no significant difference between the random and blended samples. The correlations between random and blended samples were strong (data not shown). So in this study we used weighting the random sample to quantify.

There were several limitations in our study. First of it our patients collected from two hospitals affiliated to Tongji Medical College which may result in interobserver variations. To reduce interobserver deviation, therefore, all endoscopic findings were verified by 2 gastroenterologists (Y.F and W.Y) performing endoscopic examinations over 5 years. Secondly, since our study included only small number of patients with MES 0–1 or SES-CD 0–3, it was difficult to analysis the efficiency of faecal BAFF for predicting mucosal healing in this cohort of patients, which will be explored and elucidated in our future study.

In summary, our result demonstrated that BAFF is a novel promising biomarker for differentiating IBD from IBS, and it is also a sensitive surrogate used for assessing endoscopic inflammation in IBD. The combination of faecal BAFF and calprotectin was able to increase the accuracy of differential diagnosis. Certainly, further researches will be required in our future experiments to elucidate in deep the values of faecal BAFF in IBD clinical work such as whether faecal BAFF can be used to predict relapse in IBD patients in remission, as shown in calprotectin^43^.
